# Midgut transcriptome assessment of the cockroach-hunting wasp *Ampulex compressa* (Apoidea: Ampulicidae)

**DOI:** 10.1371/journal.pone.0252221

**Published:** 2021-06-24

**Authors:** Jovana M. Jasso-Martínez, Alexander Donath, Dieter Schulten, Alejandro Zaldívar-Riverón, Manuela Sann

**Affiliations:** 1 Colección Nacional de Insectos, Instituto de Biología, Universidad Nacional Autónoma de México, Ciudad de México, Mexico; 2 Posgrado en Ciencias Biológicas, Instituto de Biología, Universidad Nacional Autónoma de México, Ciudad de México, Mexico; 3 Centre for Molecular Biodiversity Research, Zoological Research Museum Alexander Koenig, Bonn, Germany; 4 Aquazoo Löbbecke Museum, Düsseldorf, Germany; 5 Evolutionary Biology and Ecology, Institute of Biology I (Zoology), Albert-Ludwig University of Freiburg, Freiburg, Germany; USDA Agricultural Research Service, UNITED STATES

## Abstract

The emerald jewel wasp *Ampulex compressa* (Hymenoptera: Ampulicidae) is a solitary wasp that is widely known for its specialized hunting of cockroaches as larvae provision. Adult wasps mainly feed on pollen and nectar, while their larvae feed on the cockroachs’ body, first as ecto- and later as endoparsitoids. Little is known about the expression of digestive, detoxification and stress-response-related genes in the midgut of *A*. *compressa*, or about its transcriptional versatility between life stages. To identify gut-biased genes related to digestion, detoxification, and stress response, we explored the midgut transcriptome of lab-reared *A*. *compressa*, for both adults and larvae, by focusing on the top 100 significantly up- and down-regulated genes. From the top 100 significantly differentially expressed genes (DEGs), we identified 39 and 36 DEGs putatively related to digestion and detoxification in the adult wasps and larvae, respectively. The two carbohydrases alpha-glucosidase (containing an alpha-amylase domain) and glycosyl hydrolase family 31, as well as the two proteinases chymotrypsin and trypsin, revealed the highest gene diversity. We identified six significant DEGs related to detoxification, which comprise glutathione S-transferase, cytochrome P450s and UDP-glucuronosyltransferase. The gene expression levels that were significantly expressed in both life stages vary strongly between life stages, as found in genes encoding for chymotrypsin and trypsin or glycosyl hydrolases family 31. The number of genes related to alpha-glucosidase, glycosyl hydrolase family 31, and cytochrome P450s was found to be similar across nine reference hymenopteran species, except for the identified glycosyl hydrolase family 31 gene, which was absent in all reference bee species. Phylogenetic analyses of the latter candidate genes revealed that they cluster together with their homologous genes found in the reference hymenopteran species. These identified candidate genes provide a basis for future comparative genomic and proteomic studies on (ontogenetic) dietary transitions in Hymenoptera.

## Introduction

The global evolutionary success of insects is closely linked to diverse adaptations, many of which allow them to exploit and utilize various food sources. Moreover, detoxification and stress response are important adaptations to overcome chemical defenses of their food sources. As shown in other insect species, the hymenopteran midgut plays a fundamental role not only in digestion, secretion of digestive enzymes (e.g., carbohydrases, lipases, and proteinases) and absorption of nutrients, but also in the detoxification of noxious compounds and oxidative stress response [1, 2].

Stress response and detoxification of xenobiotics in insects are known to include three major and interrelated pathways: oxidation-reduction, conjugation, and hydrolysis [2, 3]. Enzymes related to oxidation-reduction comprise alcohol dehydrogenases, aldehyde dehydrogenases, cytochrome P450 monooxygenases (P450s), hydroxylases and peroxidases [2]. Cytochrome P450s in particular are known for their important role in the oxidative metabolism of endogenous compounds and xenobiotics [4, 5]. During the P450 reaction, several toxic by-products such as hydrogen peroxide, hydroxyl radicals, and superoxides are usually released. These molecules need to be further degraded by other oxidation-reduction enzymes such as catalases and peroxidases [2]. Conjugation enzymes, such as members of the superfamily glutathione S-transferases (GSTs), further catalyze the conjugation of oxidized lipids and exogenous toxins for detoxification purpose [2, 6]. Detoxification can be facilitated by hydrolytic enzymes, such as carboxyl esterases [2, 7]. Alongside their important role in stress response and detoxification, many of the above oxidation-reduction enzymes are also involved in essential physiological functions in insects [8, 9].

The biology and lifecycle of the emerald jewel wasp *Ampulex compressa* Fabr. (Hymenoptera: Apoidea: Ampulicidae) [10, 11] makes it a highly suitable model to study the expression differences of genes coding for digestive and detoxification enzymes, as well as enzymes related to oxidative stress response across life stages. This solitary species is native to the Oriental Region and, possibly Ethiopian Region and has been introduced to a number of Pacific islands, and the Neotropics [12]. The life history and famous hunting behavior of *A*. *compressa* have been documented extensively [e.g., 10, 13, 14]. Briefly, females prey on cockroaches, preferentially of *Periplaneta americana* Linnaeus as larvae provision [15, 16]. Commonly, the female wasp deposits one egg onto a mesothoracic leg of the stung and paralyzed cockroach [10, 15, 17]. The hatched larva immediately starts to feed on the cockroach’s body, first as an ecto- and later as an endo-parasitoid [10, 15, 17]. Adult wasps, in contrast, feed mainly on sugar-rich nectar or pollen. Exceptions in feeding preferences are only found in adult female wasps, which are known to show a haemolymph-drinking behavior when cutting the cockroach’s antennae during predation [14, 17–19]. Despite the large number of studies on the life history of *A*. *compressa*, little is known about its metabolic pathways and, in particular, how the expression of genes related to digestion, detoxification, and stress response change across life stages.

In this study, we examined the midgut transcriptome of lab-reared *A*. *compressa* individuals to identify gut-biased significantly differentially expressed genes (DEGs) related to digestion, detoxification, and stress response. Given the complex life history of *A*. *compressa*, we not only studied expressed genes in the midgut of adults, but also of their parasitoid larvae. Complementary DNA (cDNA) synthesized from extracted midgut RNA was sequenced on an Illumina platform, and from the top 100 significantly up- and down-regulated genes we identified those that are putatively related to digestion, detoxification, and stress response in *A*. *compressa*. We also studied the evolutionary relationships of three candidate genes coding for alpha-glucosidases, glycosyl hydrolases family 31, and cytochrome P450, for which nine genomes of representative hymenopteran species were searched for homologous gene sequences. Our results provide the first insights into the composition of gut-biased DEGs related to digestion and detoxification in *A*. *compressa*, and shed light on different expression levels between life stages.

## Materials and methods

### Sample collection

Individuals of *A*. *compressa* were obtained from long-term breeding populations at the Aquazoo Löbbecke Museum in Düsseldorf (North-Rhine Westphalia, Germany). Adult wasps were kept in 40 x 40 x 40 cm breeding boxes at 25° C equipped with a mixture of moist peat, small stones and sand. They were supplied with fresh water and a sugar-water mixture daily (50.0 g of sugar to 200 ml of water). Two cockroaches of *Periplaneta americana* were provided once a day as larvae provision. Paralyzed cockroaches carrying eggs of *A*. *compressa* were removed from the breeding boxes and transferred into a 25° C incubator for larvae development. These cockroaches were directly bred at the Aquazoo Löbbecke Museum at room temperature and fed with bananas, apples, zucchinis and fish food.

### Sample preparation

**Fig 1:**
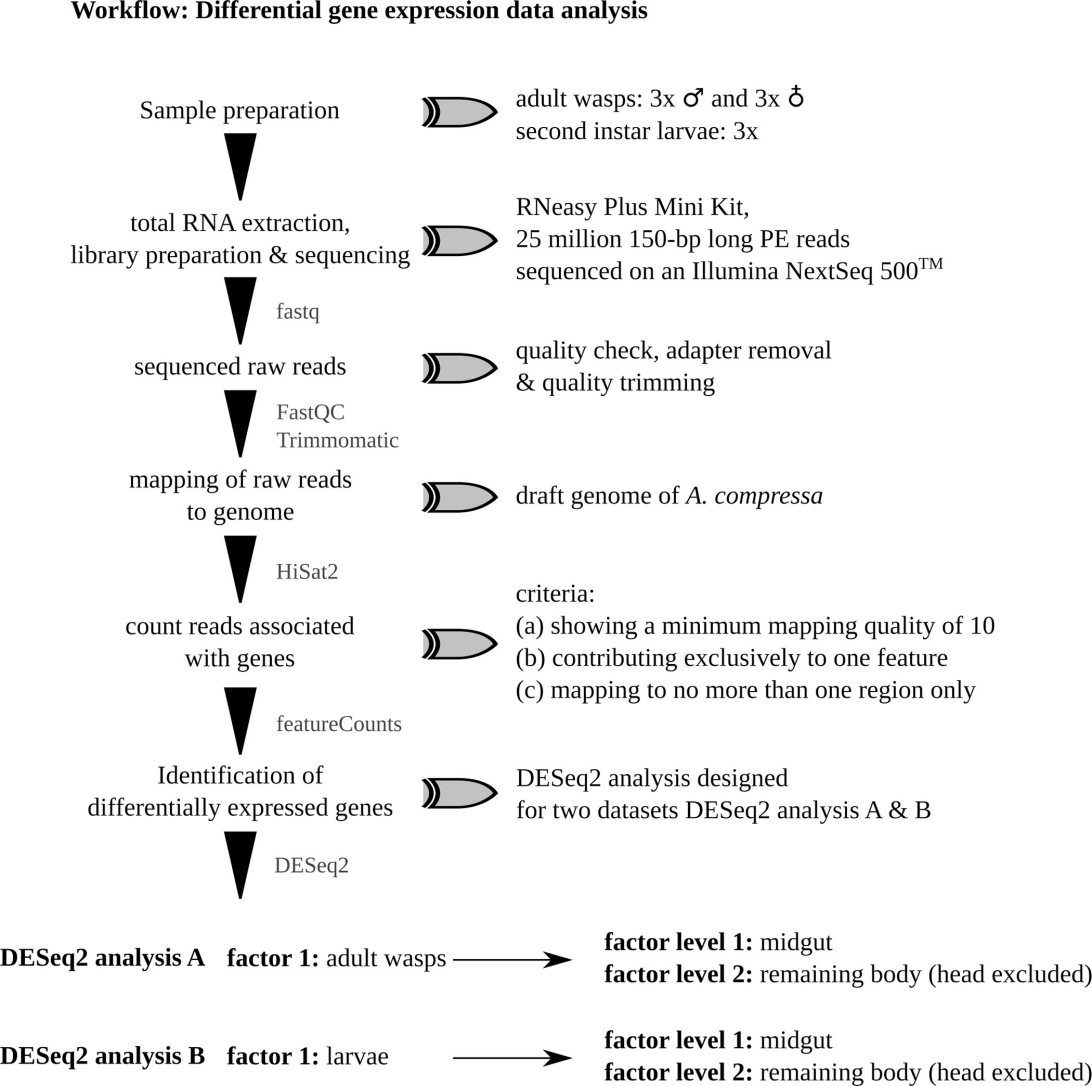
Schematic overview of the differential gene expression data analysis applied in this study. Sample preparation, RNA extraction, data checking and trimming, mapping and analysis of differential gene expression.

We sampled the midgut tissue of three independent biological replicates (three males, three females and three larvae of *A*. *compressa*) to examine the degree of variability between replicates caused by different life stages and feeding habits (Fig 1, S1 Table). The dissection of midgut tissue was performed as follows: all individuals were surface-washed with 96% pure ethanol and rinsed with distilled water. Midgut dissection was performed with sterile needles and forceps in a RNAlater^®^ (Qiagen) filled petri-dish using a binocular microscope. The midgut tissue of adult wasps was dissected by firstly cutting the abdomen, thorax and head. The abdomen was opened to dissect the midgut which was immediately stored in RNAlater. The head was removed to avoid contamination with digestive enzymes produced in the salivary glands. The remaining body parts (thorax and abdomen) were used as control samples.

Second instar larvae were carefully removed from the cockroach’s body, washed and dissected in RNAlater. The head and last segment of the body were removed, and the body was then opened by longitudinal section. At this life stage, a separation between midgut and other gut tissue is not yet developed; therefore, the whole gut tissue was included in further sample processing. The dissected gut tissue was immediately stored in RNAlater at -80°C for further preparation.

### Total RNA extraction, sequencing, and mapping

Total RNA was extracted using the RNeasy Plus Mini Kit (Qiagen, Hilden, Germany) following the manufacture’s protocol with an on-column DNAse digestion. Quality and quantity of extracted RNA was assessed with an Invitrogen^TM^ Qubit^TM^ 3.0 Fluorometer (Thermo Fischer Scientific, Waltham, USA). Extracted total RNA was stored at -80°C until library preparation. Single-indexed cDNA libraries (TruSeq stranded, dual index RNA library preparation) were paired-end (PE) sequenced with a read-length of 150-bp (25 million PE reads) on an Illumina NextSeq 500^TM^ sequencer by StarSeq GmbH in Mainz, Germany (Fig 1). Each sequencing run resulted into eight output files per sample (four forward and four reverse files), which were concatenated. The NCBI SRA accession numbers, BioProject and BioSample accessions of raw data are provided in Table 1.

**Table 1:** NCBI accession numbers, BioProject and BioSample IDs of newly sequenced and analyzed transcriptomes of *Ampulex compressa*.

Sample number	Organism name	SRA accession number	BioProject accession	BioSample accession
AC-01-TRANSC-gut	*A*. *compressa*	SRR12019114	PRJNA639361	SAMN15233319
AC-02-TRANSC-body	*A*. *compressa*	SRR12019113	PRJNA639361	SAMN15233320
AC-03-TRANSC-gut	*A*. *compressa*	SRR12019104	PRJNA639361	SAMN15233321
AC-04-TRANSC-body	*A*. *compressa*	SRR12019103	PRJNA639361	SAMN15233322
AC-05-TRANSC-gut	*A*. *compressa*	SRR12019102	PRJNA639361	SAMN15233323
AC-06-TRANSC-body	*A*. *compressa*	SRR12019101	PRJNA639361	SAMN15233324
AC-07-TRANSC-gut	*A*. *compressa*	SRR12019100	PRJNA639361	SAMN15233325
AC-08-TRANSC-body	*A*. *compressa*	SRR12019099	PRJNA639361	SAMN15233326
AC-09-TRANSC-gut	*A*. *compressa*	SRR12019098	PRJNA639361	SAMN15233327
AC-10-TRANSC-body	*A*. *compressa*	SRR12019097	PRJNA639361	SAMN15233328
AC-11-TRANSC-gut	*A*. *compressa*	SRR12019112	PRJNA639361	SAMN15233329
AC-12-TRANSC-body	*A*. *compressa*	SRR12019111	PRJNA639361	SAMN15233330
AC-13-TRANSC-gut	*A*. *compressa*	SRR12019110	PRJNA639361	SAMN15233331
AC-14-TRANSC-body	*A*. *compressa*	SRR12019109	PRJNA639361	SAMN15233332
AC-15-TRANSC-gut	*A*. *compressa*	SRR12019108	PRJNA639361	SAMN15233333
AC-16-TRANSC-body	*A*. *compressa*	SRR12019107	PRJNA639361	SAMN15233334
AC-17-TRANSC-gut	*A*. *compressa*	SRR12019106	PRJNA639361	SAMN15233335
AC-18-TRANSC-body	*A*. *compressa*	SRR12019105	PRJNA639361	SAMN15233336

Sample number from AC-01 to AC-12 represent the adult wasps and numbers from AC-13 to AC-18 the larvae.

After sequencing, raw reads were quality checked with FastQC v.0.7.2 (http://www.bioinformatics.babraham.ac.uk/projects/fastqc/). Adapter and poor-quality regions were clipped with Trimmomatic 0.32 [20] as follows. Seed mismatches: 2, palindrome clip threshold: 30, simple clip threshold: 10, minimum quality required to keep a leading base: 3, minimum quality required to keep a trailing base: 3, sliding window size: 4, required average quality in window: 15, minimum length of reads to be kept: 30.

Quality checked and trimmed raw reads were further mapped onto the available sequences of the draft genome of *A*. *compressa* (AMCO_platanus.1.2.4.n0.s3.fasta and augustus.hints.gff3) provided under Dryad repository: doi:10.5061/dryad.x69p8czj6. Further, the genome assembly and gene annotation was submitted to and quality filtered by NCBI and available under the BioProject ID: PRJNA731354 accession number JAHFVI000000000.

The genome of *A*. *compressa* was sequenced on an Illumina platform with a total coverage of 117x 150 bp paired-end (PE) reads with a 250 bp insert: 83x; 100 bp PE with an 800 bp insert PE: 16x; 100 bp mate-pair (MP) reads with a 3 kb insert 16x; 100 bp MP with an 8 kb insert: 2x; largest scaffold: 16.3 Mbp, scaffold N50: 9.13 Mbp, scaffold L50: 12. The genome was sequenced and annotated by the Leibniz Graduate School on Genomic Biodiversity Research (GBR) of the Zoological Research Museum Alexander Koenig (ZFMK) (Bonn, Germany), and it was provided by Prof. Dr. Bernhard Misof.

Mapping of raw reads onto the genome was performed with the software HISAT2 2.1.0 using default settings [21]. Individual read count tables were subsequently generated with featureCounts (part of the Subread 1.6.3 software package [22]). Mapped reads were counted when (a) showing a minimum mapping quality of 10, (b) contributing exclusively to one feature (*i*.*e*., genomic regions such as genes, exons, promoters and genomic bins), and (c) mapping to no more than one region (Fig 1).

### Analysis of differential gene expression

We performed a differential gene expression (DGE) analysis with DESeq2 v.2.11.40.6+galaxy1 [23] implemented in the web-based scientific analysis platform Galaxy (https://galaxyproject.org [24]) to determine gut-biased DEGs from the generated count tables. The DESeq2 analysis applies shrinkage estimation for dispersion and fold changes [23] and performs a median of ratios method by default [23] to normalize sequencing depth and RNA composition.

We performed two separated DESeq2 analyses to identify gut-biased DEGs: (A) analyzing the transcriptomes of the adult wasps by comparing midgut *vs*. remaining body (head excluded) and (B) analyzing the transcriptomes of the larvae comparing midgut *vs*. remaining body (Table 2, Fig 1). We kept all genes with a significant adjusted *p*-value below 0.05 and an absolute fold change (FC) > 2 to extract candidate genes with a significant change in gene expression. Subsequently, we focused on the top 100 significantly up-regulated and down-regulated DEGs. We mapped all identified up-regulated candidate genes against the reference gene annotation file of *A*. *compressa* to identify the location of DEGs within the genome.

**Table 2:** DESeq2 design specified for DGE analysis in this study.

**DESeq2 analysis A**	**Factor 1** adult wasps	**Factor level 1**	**Factor level 2**
		midgut	remaining body (head excluded)
**DESeq2 analysis B**	**Factor 1** larvae	**Factor level 1**	**Factor level 2**
		midgut	remaining body (head excluded)

Factor 1 of the DESeq2 analysis A “adult wasps” comprises a total of twelve transcriptome samples i.e., six “midgut” and six “remaining body” individual samples. Factor 1 of DESeq2 analysis B “larvae” comprises a total of six transcriptome samples i.e., three “midgut” and three “remaining body” individual samples.

### Functional annotation and gene ontology

We performed a blastp search on all up-regulated genes against the non-redundant protein database in the Basic Local Alignment Search Tool (BLAST) v.2.2.31+ [25, 26] with the following parameters: taxonomic filter: Insecta, e-value: 1E-5, and word size = 3. Additionally, InterProScan was run on all protein sequences (options -f tsv -t p -pa -goterms, version 5.35.74.0) to classify protein sequences into protein families [27, 28]. Finally, we searched against the eggNOG database version 5.0 [29]. Genes were considered as candidate genes only if matching a BLAST hit and additionally exhibiting both, a gene ontology term (GO) [30] and an eggNOG annotation. All candidate DEGs were classified based on whether they have a well-known function in digestion, detoxification or stress response. To maintain only trusted trypsins and/or chymotrypsins, all identified protein sequences were searched for the presence of the catalytic triad (aspartate, histidine and serine) that is crucial for its specificity ([31]; S2 Table).

### Comparing the number of putative glucosidase and P450 candidate genes identified in *A*. *compressa* across other Hymenoptera

To assess differences in the number of genes related to digestion and detoxification across various aculeate species, we searched the protein sequences of three identified carbohydrases (enzyme code (EC): 3.2.1.20) and three cytochrome P450 (EC: 1.14) candidate genes against the GenBank database at the National Center for Biotechnology Information (NCBI) (http://www.ncbi.nlm.nih.gov/BLAST/; [32]). The BLAST protein search was restricted with a cut-off e-value of 10^−5^ and to the following representative hymenopteran species with well-annotated genome and different feeding habits: three bee species *Apis mellifera* (taxid: 7460; assembly Amel_HAv3.1) [33], *Dufourea novaeangliae* (taxid: 178035, assembly ASM127255v1; http://i5k.github.io), and *Megachile rotundata* (taxid: 143995; assembly MROT_1.0) [34], one vespid wasp *Polistes canadensis* (taxid: 91411, assembly ASM131383v1; http://i5k.github.io), two ant species *Acromyrmex echinatior* (taxid: 103372) [35] and *Camponotus floridanus* (taxid: 104421; assembly Cflo_v7.5) [36], one chalcid wasp species *Nasonia vitripennis* (taxid:7425; assembly Nvit_psr_1.1) [37], and two sawfly species *Athalia rosae* (taxid: 37344, assembly Aros_2.0; http://i5k.github.io) and *Orussus abietinus* (taxid: 222816; assembly Oabi_2.0) [38].

We performed a reciprocal blast search on the three best hits per candidate gene found for each reference species by applying the following parameters: [-d] = genome of *A*. *compressa*, [-p] = tblastp, [-e] = 10^−5^, [-m8], and [-i] = reference protein sequence. The same search parameters were applied to the selected candidate genes found in *A*. *compressa*. Only the top two hits were retained and counted as reliable matches. Genes found for each reference species that matched the same scaffold and the same or similar position in the genome of *A*. *compressa* as the respective candidate genes were retained and counted as reliable matches. Finally, all identified candidate genes and reference protein sequences were searched against the pfam database [39].

### Gene tree inferences

We inferred phylogenetic trees from the three carbohydrases (EC: 3.2.1.20) and cytochrome P450 (EC: 1.14) gene sequences that were identified in *A*. *compressa* and of their homologous gene sequences identified across nine reference Hymenoptera species (S7 and S8 Tables).

Amino acid sequence alignments were generated with MAFFT version 7 [40] using the iterative refinement method FFT-NS-i with the default parameters. We selected the best-fit substitution model according to the Bayesian Information Criterion with ModelFinder [41] implemented in IQTREE version 2 [42]. We conducted two Maximun Likelihood (ML) analyses using (1) the glucosidases matrix (28 terminals, 645 aminoacid positions) and (2) the P450 matrix (49 terminals, 904 aminoacid positions) in IQ-TREE version 2 [42] with 1,000 ultra-fast bootstrap (UFB) replicates.

## Results

### Transcriptome sequencing and data processing

We generated between 33,080,708 and 52,495,398 paired-end raw sequencing reads per sample, which yielded 15,721,379 to 25,592,150 reads pairs after adapter and low quality sequence trimming (S3 Table). Mapping of raw reads onto the genome of *A*. *compressa* resulted in mapping rates between 74.9% and 98.0% per sample (S3 Table). Between 47.2% and 76.6% of the quality-checked and trimmed reads were unambiguously assigned to an annotated feature (*i*.*e*., genomic regions such as genes or exons; S3 Table).

### Differential gene expression (DGE) analyses

**Fig 2:**
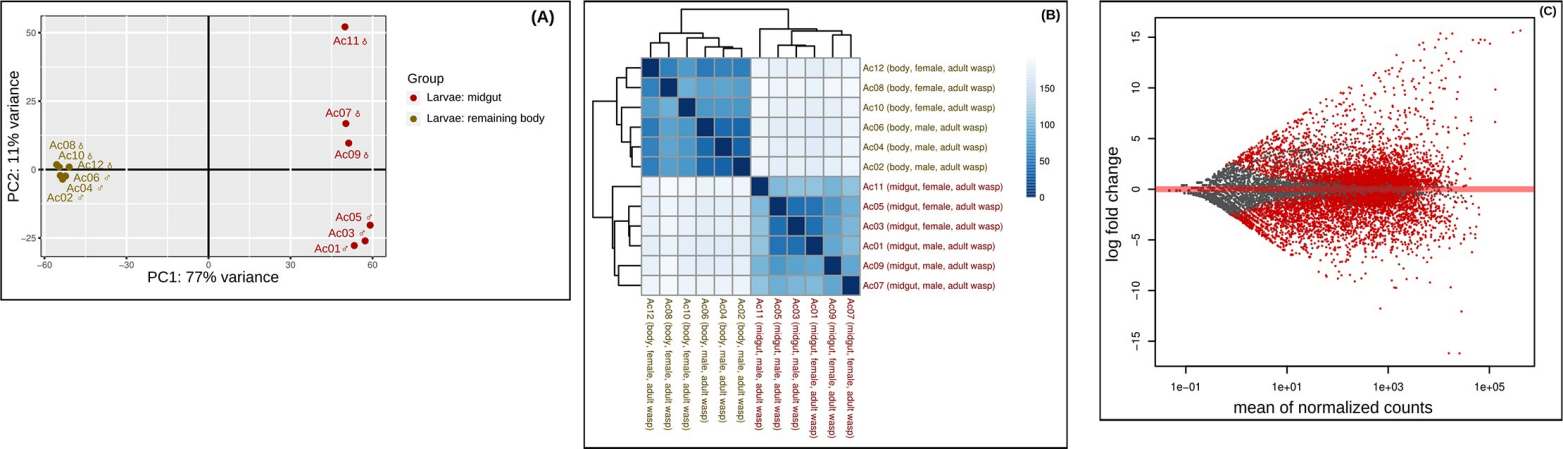
Principal component analysis, heatmap and MA plot. (**A**) Principal component analysis (PCA) of all genes with normalized counts. The plot shows the distribution of the 12 adult wasps transcriptome samples analyzed in the DESeq2 analysis A. The first dimension separates samples by body part *i*.*e*., midgut vs. remaining body, and the second separates samples by sex *i*.*e*., female and male adult wasps. (**B**) Heatmap of the sample-to-sample distances based on the normalized counts. Samples are grouped by body part (midgut *vs*. remaining body) as shown in the PCA plot. Color represents distance between the samples. Dark blue means shorter distance. (**C**) MA plot of the log2 fold change of all genes. Genes that passed the significance threshold (adjusted *p*-value < 0.1) are colored in red.

**Fig 3:**
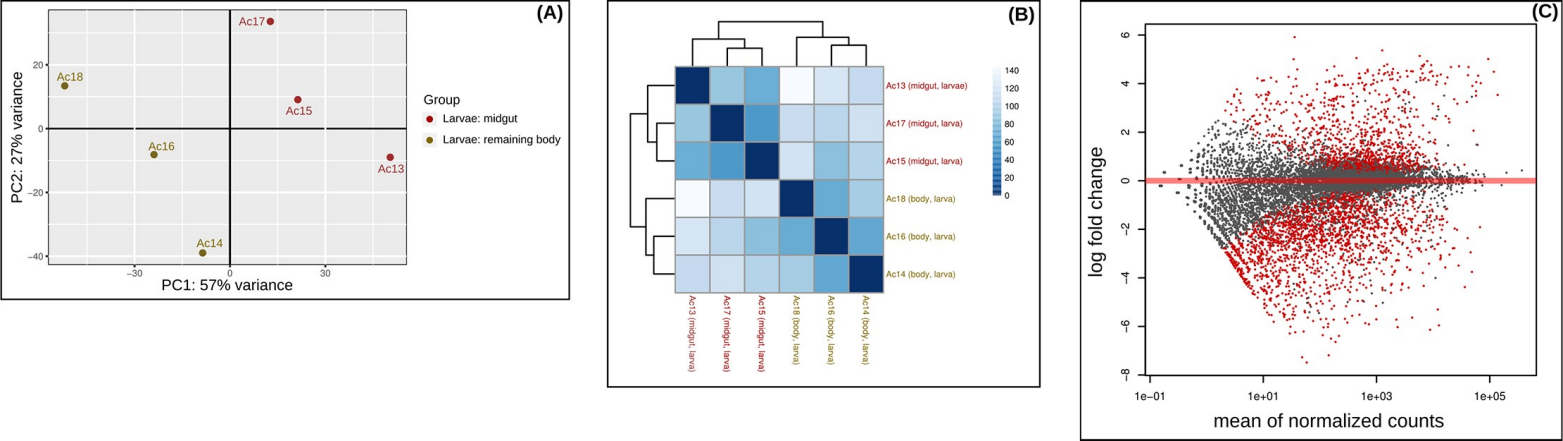
Principal component analysis, heatmap and MA plot. (**A**) Principal component analysis (PCA) of all genes with normalized counts. The plot shows the distribution of the six larvae transcriptome samples analyzed in the DESeq2 analysis B. The first dimension separates samples by body part *i*.*e*., midgut vs. remaining body. (**B**) Heatmap of the sample-to-sample distances based on the normalized counts. Samples are grouped by body part (midgut vs. remaining body) as shown in the PCA plot. Color represents distance between the samples. Dark blue means shorter distance. (**C**) MA plot of the log2 fold change of all genes. Genes that passed the significance threshold (adjusted p-value < 0.1) are colored in red.

We performed two separated DGE analyses to identify gut-biased DEGs with a putative role in digestion, detoxification and oxidative stress response in the transcriptome of *A*. *compressa* (Figs 1–3). The DESeq2 analysis A (adult wasps: midgut vs. remaining body) resulted into 19,080 DEGs, which dropped, after filtering for a significant change in gene expression and an absolute fold change (FC) > 2, to a total of 4,913 candidate genes (Table 3 and S4 Table). The top 100 up-regulated DEGs span a log2(FC) of 15.6 to 7.8 and the top 100 down-regulated DEGs span a log2(FC) of -16.2 to -5.7 (S4 Table). For the down-stream analyses, the candidate DEGs were considered as gut-biased. The PCA shows a distribution with two principal dimensions: (1) gut-biased and body-biased DEGs, which explain 77% of the variance and (2) female- and male-biased DEGs, which are explained by only 11% of the variance (Fig 2).

**Table 3:** Results of the DESeq2 data analyses performed in this study.

Analysis step	DESeq2 analysis A	DESeq2 analysis B
Determined differentially expressed genes (DEGs)	19,080	19,080
Extracted genes with a significant change in gene expression (adjusted *p*-value below 0.05)	7,566	2,629
Extracted genes with an absolute fold change (FC) > 2	4,913	2,084

The table presents the partial results on the number of identified genes that resulted from each data analyses step. For more details, please refer to the S4 Table.

The DESeq2 analysis B (larvae: midgut vs. remaining body) resulted into 19,080 DEGs, which dropped after filtering for a significant change in gene expression and an absolute fold change (FC) > 2 to a total of 2,084 candidate genes (Table 3 and S4 Table). The top 100 up-regulated DEGs span a log2(FC) of 5.9 to 3.8 and the top 100 down-regulated DEGs span a log2(FC) of -7.5 to -4.6 (S4 Table). The PCA shows that the dataset separates according to gut-biased and body-biased DEGs, which is explained by 57% of the variance (Fig 3). All results of read mapping, read count tables and the DGE analyses are available at Dryad Repository https://doi.org/10.5061/dryad.x69p8czj6.

### Candidate genes related to digestion, detoxification, and oxidative stress response

From the top 100 DEGs that are considered as gut-biased, we identified 39 and 36 DEGs putatively related to digestion and detoxification in the adult wasps and larvae, respectively (S5 and S6 Tables). We found DEGs coding for 17 carbohydrases, 43 proteinases, nine lipases, and six detoxification enzymes in both life stages (S5 and S6 Tables). All identified trypsin- and chymotrypsin-like genes were confirmed according to their characteristic catalytic triad histidine, aspartate, and serine [43] (S2 Table).

In the midgut transcriptome of the adult wasps, we identified eight carbohydrases (comprising two down- and six up-regulated genes), 23 proteinases (comprising two down- and 21 up-regulated genes), four lipases (comprising one down- and three up-regulated genes) and four detoxification enzymes (comprising one down- and three up-regulated genes) (Tables 4 and 5, S5 Table). In the midgut transcriptome of the larvae, we identified nine carbohydrases (comprising up-regulated genes only), 20 proteinases (comprising four down- and 16 up-regulated genes), five lipases (comprising three down- and two up-regulated genes), and two detoxification enzymes (comprising up-regulated genes only) (Tables 6 and 7, S6 Table). All identified sequences of up- and down-regulated genes are available at Dryad Repository https://doi.org/10.5061/dryad.x69p8czj6.

**Table 4:** Candidate genes putatively involved in digestion identified in the transcriptome of *A*. *compressa* adult wasps.

Enzyme group	Gene ID	Putative enzyme
Carbohydrases	g1074, g3038, g10797,	sugar (and other) transporter
	g2792	alpha-amylase-like (Aamy_C)
	g12463, g16664, g16665	alpha-amylase domain
	g13972	glycosyl hydrolases family 31
Lipases	g7768, g8153, g9027	pancreatic triacylglycerol lipase-like
	g15485	elongation of very long chain fatty acids protein
Proteinases	g1997	serine carboxypeptidase
	g3419, g3420, g15842	metallocarboxypeptidase (Zn_pept)
	g4146	aminotransferase class I and II
	g5100, g5101, g5103, g8318, g8320, g8321, g9870, g11873, g11875, g11876, g14305, g14306, g14307, g14309, g16229, g18034, g18035, g18036	chymotrypsin- or trypsin-like

Given are up- and down-regulated differentially expressed genes (DEGs; Gene ID) with a putative function in digestion. Underlined gene IDs were found as down- and non-underlined gene IDs as up-regulated DEGs. For a more detailed information please refer to the S5 Table in the Supplementary information material.

**Table 5:** Candidate genes putatively involved in detoxification identified in the transcriptome of *A*. *compressa* adult wasps.

Enzyme group	Gene ID	Putative enzyme
P450s	g8966, g14510, g15273	cytochrome P450
GSTs	g16774	glutathione S-transferase

Given are up- and down-regulated differentially expressed genes (DEGs) with a putative function in detoxification. Underlined gene IDs were found as down- and non-underlined gene IDs as up-regulated DEGs. Detailed information can be found in the S5 Table.

**Table 6:** Candidate genes putatively involved in digestion identified in the transcriptome of *A*. *compressa* larvae.

Enzyme group	Gene ID	Putative enzyme
Carbohydrases	g255	alpha-mannosidase
	g2196, g2197	alpha-L-fucosidase
	g2791	alpha-amylase (Amyrel)
	g4615, g9602	glycosyl hydrolases family 2
	g10797, g18815	sugar (and other) transporter
	g13972	glycosyl hydrolases family 31
Lipases	g3507	lipase 3-like
	g7768	pancreatic triacylglycerol lipase-like
	g12671, g14610, g15485	elongation of very long chain fatty acids protein
Proteinases	g1997	serine carboxypeptidase
	g2325	metallopeptidase (ERAP1-like)
	g4146	aminotransferase class I and II
	g4238, g5101, g5102, g5103, g8320, g9871, g11875, g11876, g14305, g14306, g14307, g14308, g14310, g14312, g16229, g18720	chymotrypsin- or trypsin-like
	g18807	ERAP1-like C-terminal domain

Given are up- and down-regulated differentially expressed genes (DEGs) with a putative function in digestion. Underlined gene IDs were found as down- and non-underlined gene IDs as up-regulated DEGs. Detailed can be found in the S6 Table.

**Table 7:** Candidate genes putatively involved in detoxification identified in the transcriptome of *A*. *compressa* larvae.

Enzyme group	Gene ID	Putative identification
P450s	g10492	cytochrome P450
UDPGs	g5346	UDP-glucuronosyltransferase

Given are up-regulated differentially expressed genes (DEGs) with a putative function in detoxification. Detailed information can be found in the S6 Table.

### Differential gene expression between life stages of *A*. *compressa*

To study gut-biased significantly expressed genes related to digestion, detoxification, and stress response in the transcriptome of *A*. *compressa*, we sampled six adult wasps (three males and three females) and three larvae. Despite the relatively small sampling size, some indications on DEGs between the two observed life stages of *A*. *compressa* can be made. All annotated and significant DEGs were searched against the eggNOG database, but we refrained from performing further statistical comparisons on the functional categories. The top 100 identified up- and down-regulated DEGs were assigned to three eggNOG classifications, comprising information storage and processing, cellular processes and signaling, and metabolism (Table 8).

**Table 8:** EggNOG results on the orthology relationships, gene evolutionary histories and functional annotations of the top 100 gut-biased DEGs found in the transcriptome of *A*. *compressa*.

eggNOG categories	Adult wasps		Larvae	
	up	down	up	down
**Information storage and processing**				
Transcription	6	5	4	7
Translation, ribosomal structure and biogenesis	0	0	0	0
Replication, recombination and repair	0	0	0	0
RNA processing and modification	0	1	0	1
Chromatin structure and dynamics	0	1	0	0
**Cellular processes and signaling**				
Posttranslational modification, protein turnover, chaperones	25	4	17	8
Signal transduction mechanisms	3	7	6	10
Intracellular trafficking, secretion, and vesicular transport	3	1	1	0
Cell wall/membrane/envelope biogenesis	2	2	1	0
Extracellular structures	1	0	0	1
Cytoskeleton	0	2	0	0
Cell cycle control, cell division, chromosome partitioning	0	0	2	1
Nuclear structure	0	0	0	0
Defense mechanisms	0	0	0	0
Cell motility	0	0	0	0
**Metabolism**				
Carbohydrate transport and metabolism	8	3	12	0
Inorganic ion transport and metabolism	6	1	6	2
Lipid transport and metabolism	4	2	6	6
Amino acid transport and metabolism	3	1	3	6
Secondary metabolites biosynthesis, transport and catabolism	4	3	3	3
Nucleotide transport and metabolism	1	0	0	0
Energy production and conversion	0	0	2	0
Coenzyme transport and metabolism	0	0	0	0
**Poorly characterized**				
Function unknown (S)	14	16	18	23
General function prediction only (R)	0	0	0	0
**General information**				
Total amount of input sequences	100	100	100	100
Average length	481.0	384.0	462.0	502.0
Number of GO annotated sequences	42	24	37	24
Number of GO annotations	224	166	180	165
Average GOs per sequence	5.33	6.92	4.86	6.88

The table summarizes all annotations obtained on the top 100 gut-biased DEGs that could be transferred with EggNOG Mapper. Given are up- and down-regulated DEGs identified in both life stages (adult wasps and larvae). The columns provide the number of sequences found as up-regulated genes “up” and down-regulated genes “down” identified in both life stages of *A*. *compressa* that clustered with the different orthologous groups.

**Fig 4:**
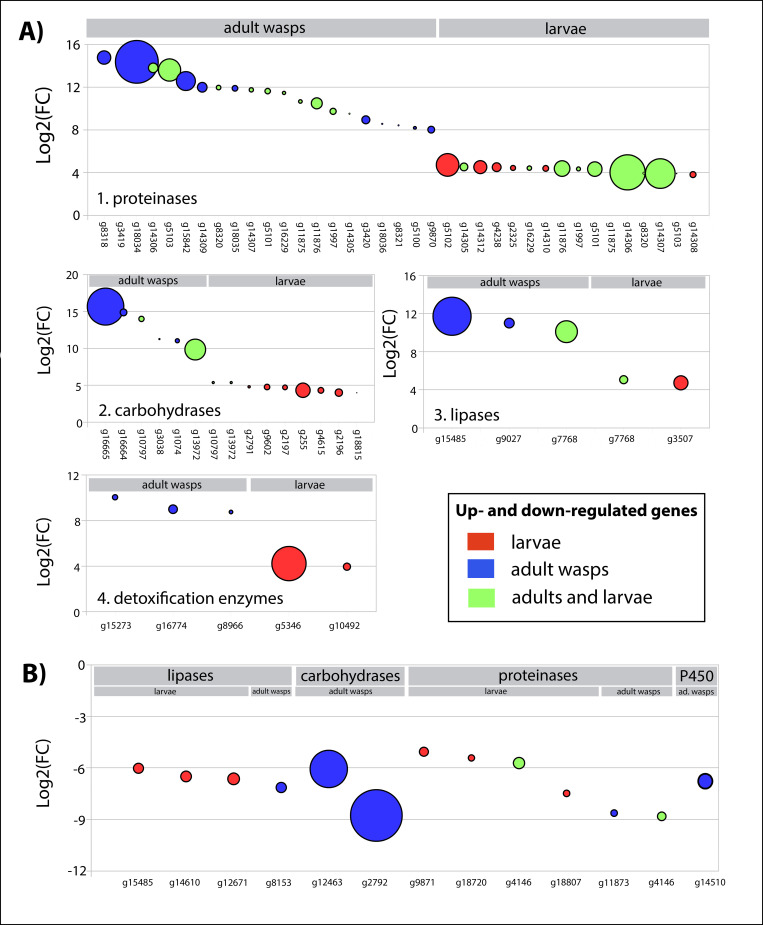
Normalized expression level of up- and down regulated genes putatively involved in digestion and detoxification processes. **A)** Up-regulated genes identified in the midgut transcriptome of *A*. *compressa* adult wasps and larvae (grey bars) are grouped according to their coding function as 1) proteinases, 2) carbohydrases, 3) lipases, and 4) detoxification enzymes. **B)** Down-regulated genes identified in the midgut transcriptome of *A*. *compressa* adult wasps and larvae (grey bars) are grouped according to their coding function as 1) proteinases, 2) carbohydrases, 3) lipases, and 4) detoxification enzymes (grey bars). Up- and down-regulated genes (x-axis) are plotted against their log2(FC) (y-axis). Genes exclusively found either in the adult wasps or larvae are highlighted in blue and red, respectively. Genes found in both life stages are highlighted in green. Circle size represents the number NRC found for the respective gene. Detailed information on candidate up- and down-regulated genes is provided in S5 and S6 Tables.

Several gut-biased up- and down-regulated DEGs show a specific high expression, *i*.*e*. a higher mean of normalized read counts (NRC). Conspicuous high expression levels were found in four up-regulated genes in the adult wasps: g13972 and g16665 coding for the two carbohydrases glycosyl hydrolases family 31 and alpha-amylase, g18034 coding for the proteinase serine 9-like, and g15485 encoding a lipase GNS1/SUR4 family. The genes g14306 and g14307, coding for typsin and chymotrypsin respectively, exhibit high means of NRC in the larvae (Fig 4). Moreover, the two down-regulated genes g12463 and g2792 found in adults wasps coding for the carbohydrases alpha-amylase and alpha-glucosidase respectively, exhibit a conspicuous high mean of NRC (Fig 4).

### Comparing the number of selected candidate genes identified in *A*. *compressa* across other Hymenoptera

To obtain a first impression of the diversity of digestion and detoxification related genes across different Hymenoptera species, we searched three selected candidate genes coding for carbohydrases (alpha-glucosidases and glycosyl hydrolases family 31; EC: 3.2.1.20) and three coding for cytochrome P450 (EC: 1.14) that were identified in *A*. *compressa* against nine reference species using the NCBI refseq-protein database. When searching for the three glucosidase genes, we received between ten (*D*. *novaeanglia*) and 24 (*C*. *floridanus*) homologous genes and when searching for the three cytochrome P450 candidate genes, we received and between 38 (*O*. *abietinus*) and 179 (*C*. *floridanus*) (Table 9).

**Table 9:** BLAST homologous hit table.

Enzyme(s) and number of selected candidate genes identified in *A*. *compressa*	*Polistes canadensis*	*Apis mellifera*	*Dufourea novaeangliae*	*Megachile rotundata*	*Acromyrmex echinatior*	*Camponotus floridanus*	*Nasonia vitripennis*	*Athalia rosae*	*Orussus abietinus*
**digestive enzyme genes**									
Glucosidases (3)	16	15	10	21	17	24	18	20	14
**detoxification genes**									
Cytochrome P450 (3)	63	66	46	61	79	179	108	67	38

Selected gut-biased candidate genes putatively involved in digestion (glucosidases, EC: 3.2.1.20) and detoxification (cytochrome P450, EC: 1.14) identified in *A*. *compressa* blasted against the NCBI refseq-protein database with a cut-off E-value of 10^−5^. The BLAST-search was restricted to nine representative Hymenoptera species with well-known and annotated genome. Shown are the numbers of homologous hits found for the respective species.

We further searched the top three BLAST hits against the genome of *A*. *compressa* to assess the variation of glucosidase and cytochrome P450 genes across other Hymenoptera. Only if the first two resulting hits corresponded with the same scaffold and the same or similar position in the genome of *A*. *compressa*, as found for the respective candidate gene(s) in the *A*. *compressa* transcriptome, they were counted as a significant match (Table 10 and S7 and S8 Tables). The gene diversity for the identified glucosidase alpha-amylase in *A*. *compressa* is similar to that of all other Hymenoptera species, with *C*. *floridanus* and *N*. *vitripennis* exhibiting the highest gene variation (Table 10 and S7 Table). The diversity of glycosyl hydrolases family 31 genes varied across all Hymenoptera species (Table 10 and S7 Table). While most species exhibit one gene copy (*A*. *compressa*, *P*. *canadensis*, *A*. *echinatior*, and *O*. *abietinus*), the parasitic wasp *N*. *vitripennis* and the ant *C*. *floridanus* exhibit three gene copies (Table 10 and S7 Table). We did not find glycosyl hydrolases family 31 genes that could correspond with the *A*. *compressa* gene in all bee species (Table 10 and S7 Table). The variation of cytochrome P450 genes found in the transcriptome of *A*. *compressa* is similar to that of all other hymenopteran species (Table 10 and S8 Table). The lowest number of cytochrome P450 gene copies that correspond with the *A*. *compressa* genes was found for the parasitic wasp *N*. *vitripennis* (Table 10, S8 Table).

**Table 10:** Significant matches of identified glucosidase and cytochrome P450 gene copies found in *A*. *compressa* and other Hymenoptera species.

Enzymes vs. number of identified gene copies	*Ampulex compressa*	*Polistes canadensis*	*Apis mellifera*	*Dufourea novaeangliae*	*Megachile rotundata*	*Acromyrmex echinatior*	*Camponotus floridanus*	*Nasonia vitripennis*	*Athalia rosae*	*Orussus abietinus*
Alpha-glucosidases with an alpha-amylase catalytic domain	**2**	4	3	3	2	3	5	6	5	3
Glycosyl hydrolases family 31	**1**	1	0	0	0	1	3	3	3	1
Cytochrome P450	**3**	2	4	3	3	4	3	1	3	3

Shown are the enzyme names according to the pfam description *vs*. the number of identified gene copies when searching the top three Blast hits against the genome of *A*. *compressa*. Only the top two hits were counted as significant match when matching the same scaffold and the same or similar position in the genome of *A*. *compressa* as found for the respective candidate genes in the midgut transcriptome of *A*. *compressa*. Detailed information is provided in the S7 and S8 Tables.

### Gene trees

**Fig 5:**
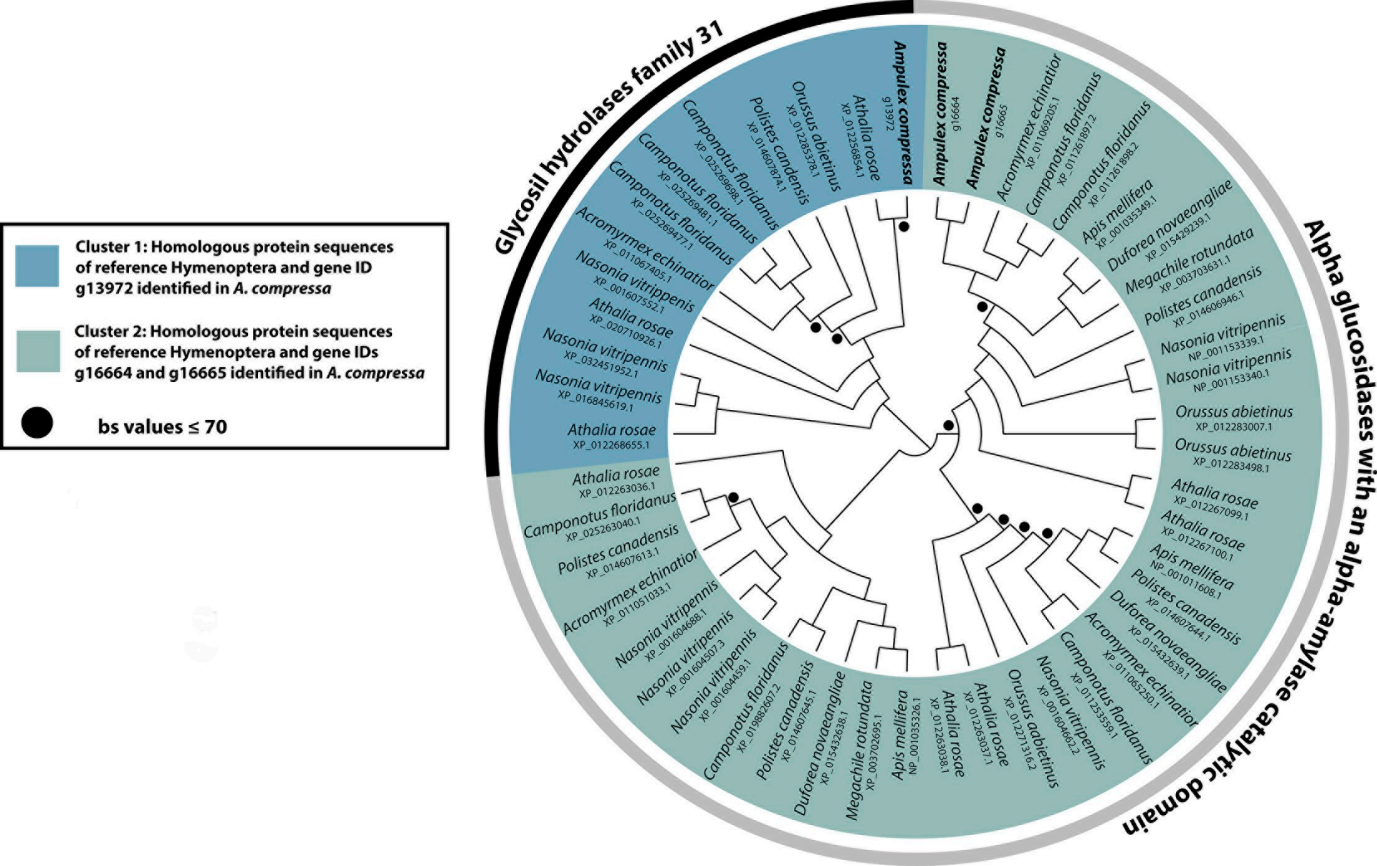
Unrooted maximum likelihood tree of candidate alpha-glucosidase protein sequences identified in the midgut transcriptome of *A*. *compressa* and nine reference Hymenoptera species. The two main groups recovered are labeled in the tree. One comprises glycosyl hydrolase family 31 and the other all alpha-glucosidase gene sequences that are characterized with an alpha-amylase catalytic domain. Black dots indicate bootstrap support values ≤ 70. All protein sequences of *A*. *compressa* are available as Supplementary data at Dryad Repository. Accession numbers and information on the reference taxa are provided in the S7 Table.

The phylogenetic analysis of the three putative alpha-glucosidase candidate genes (EC: 3.2.1.20) identified in *A*. *compressa* and all 46 homologous gene copies identified in the nine Hymenoptera reference taxa resulted in two main groups. One group comprises all glycosyl hydrolase family 31 gene sequences, and the second all alpha-glucosidase gene sequences that are characterized with an alpha-amylase catalytic domain (S7 Table, Fig 5). The latter group is further divided into three subgroups (Fig 5).

**Fig 6:**
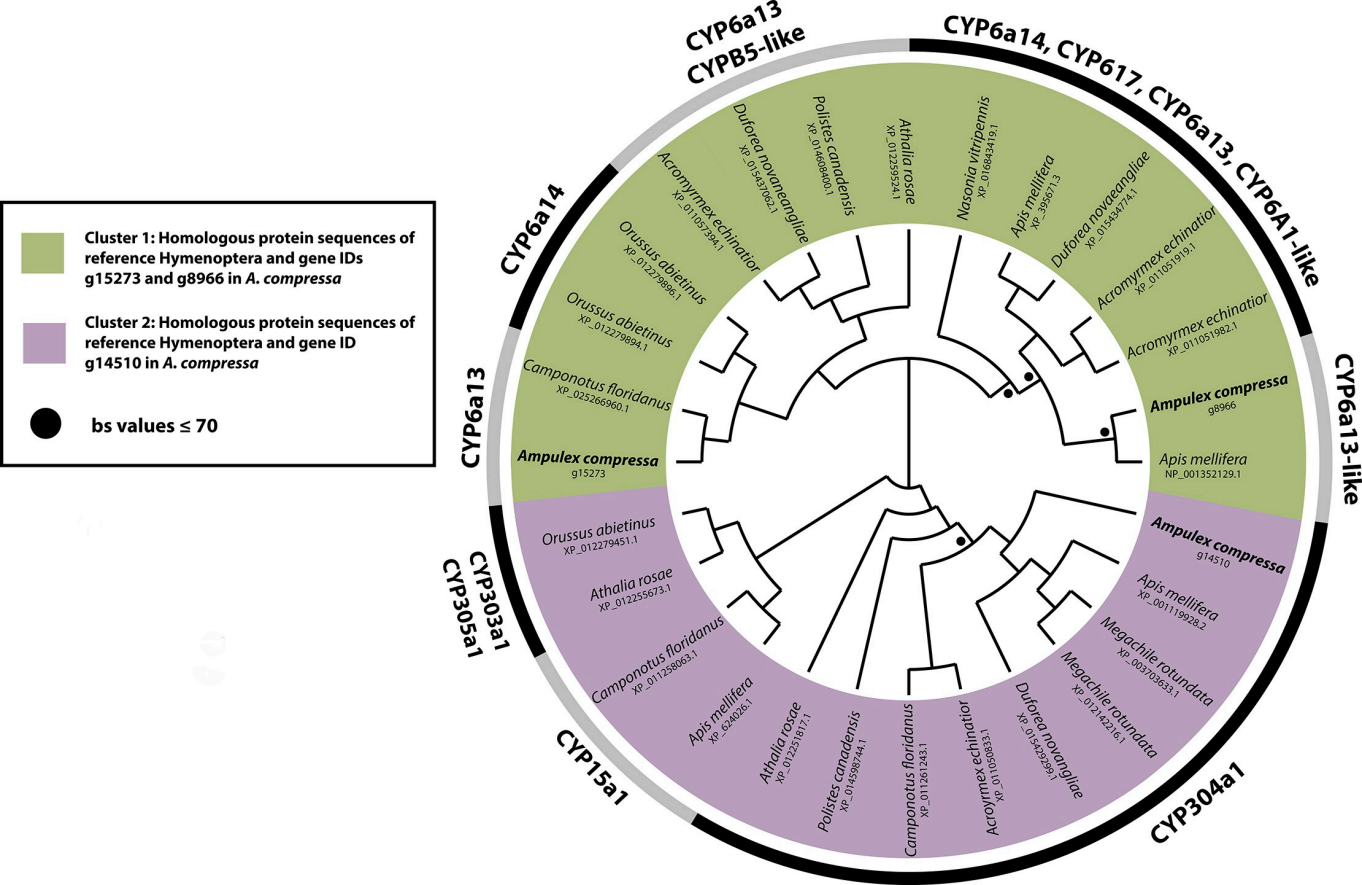
Unrooted maximum likelihood tree of candidate cytochrome P450 protein sequences identified in the midgut transcriptome of *A*. *compressa* and nine reference Hymenoptera species. The two main groups and four subgroups recovered are labeled in the tree. Black dots indicate bootstrap support values ≤ 70. All protein sequences of *A*. *compressa* are available as Supplementary data at Dryad Repository. Accession numbers and information on the reference taxa are provided in the S8 Table.

The phylogenetic analysis of the three cytochrome P450 candidate genes (EC: 1.14) identified in *A*. *compressa* and all 25 homologous gene copies identified in the nine Hymenoptera reference taxa resulted in two main groups. The first main group comprises all cytochrome P450 gene sequences that correspond to the CYP6 family represented by the subfamilies CYP6a13, CYP6a14, CYP6a17, CYP6a1-like, and CYP6B5-like (Fig 6). The second main group is further divided into three subgroups, one comprising CYP303a1 and CYP305a1, a second CYP15a1 and the remaining one all CYP304a1 gene sequences (Fig 6).

## Discussion

In this study, we examined the transcriptome of the jewel wasp *A*. *compressa* across two life stages with notably different feeding habits. We identified gut-biased up- and down-regulated genes with a fundamental role in digestion and detoxification (Tables 3–7 and S5 and S6 Tables), and compared our results between the two life stages. Further, we provide insights on the gene number and diversity of alpha-glucosidases, glycosyl hydrolase family 31, and cytochrome P450 related candidate genes found differentially expressed in the midgut transcriptome of *A*. *compressa* compared with other representative Hymenoptera species.

### Candidate genes related to digestion, detoxification, and oxidative stress response

We identified significant DEGs in the midgut transcriptome of *A*. *compressa* by analyzing individuals from two fundamentally different life stages. Thus, we expect to provide a comprehensive overview on digestive and detoxification genes in the midgut transcriptome of *A*. *compressa*. Studies on the midgut transcriptome in Hymenoptera are sparse; however, studies analyzing the whole transcriptome in response to digestion, detoxification and stress response are more common (*e*.*g*., in *Bombus huntii* [2], in the fire ant *Solenopsis invicta* [44], in *Meteorus pulchricornis* [45], or in the honeybee *Apis mellifera* [46]).

The principal site of digestion, secretion of digestive enzymes and absorption of nutrients is the midgut [1]. However, food absorbed and chewed through the mouth is already pre-processed and digested by enzymes released by salivary glands. Furthermore, microbial gut communities are important for nutrition and pathogen resistance in many living species. In this study, we identified 15 carbohydrases, eight lipases, and 32 proteinases in the midgut transcriptome of *A*. *compressa* (Tables 4 and 6 and S5 and S6 Tables).

The most common carbohydrase comprises alpha-glucosidases, glycosyl hydrolase family 31 (EC: 3.2.1.20) and sugar (and other) transporter (Tables 4 and 6). All identified alpha-glucosidases are characterized by an alpha-amylase catalytic domain, and thus belong to the alpha-amylase family (glycoside hydrolase family 13). These glycosyl hydrolases mainly catalyze the hydrolysis of glycogen and starch, whose products are further hydrolyzed to glucose [47]. Alpha-amylases in particular are of great importance for nutrition in all living organisms, and thus are represented by a different number of gene copies in the genome [48]. In Hymenoptera, the reported gene number varies from three in *A*. *mellifera* (Hbg1, Hbg1, and Hbg3) to six in *N*. *vitripennis* [47].

The number of alpha-amylase gene copies cannot be clearly linked to the dietary habit; however, dietary flexibility might increase with the number of gene copies [48]. In our study, we identified two gene copies of an alpha-glucosidase with an alpha-amylase catalytic domain in *A*. *compressa* (Table 10). When comparing these gene copies across nine representative Hymenoptera (Table 10), the parasitoid wasp *N*. *vitripennis* exhibits the highest gene diversity with six corresponding gene copies, followed by five gene copies found in the genome of the omnivorous ant *C*. *floridanus* and the turnip sawfly *A*. *rosae* (Table 10). No clear pattern in the number of gene copies and feeding habits can be found across the chosen reference species. The higher number of gene copies found in *A*. *rosae* might be a hint to its relationship to plants during larval development, since higher amylase activities are described for phytophagous species [49]. Interestingly, the identified enzyme glycosyl hydrolase family 31, represented by one gene in the midgut transcriptome of *A*. *compressa*, could not be confirmed in the genome of any bee species included as reference in this study, but was found in multiple copies in *A*. *rosae*, *C*. *floridanus*, and *N*. *vitripennis* (Table 10).

The detoxification enzyme cytochrome P450 plays a major role in the oxidative transformation of endogenous and exogeneous molecules in all living organisms [50]. High expression levels of cytochrome P450s are reported to allow insects to metabolize diverse insecticides and other xenobiotics, thereby enabling insecticide resistance [3]. Here we found three P450 candidate genes of which two were up- and one was down-regulated in the midgut transcriptome of *A*. *compressa* (Tables 5 and 7). Similar corresponding gene copies can be found across other Hymenoptera species (Tables 9 and 10). Along with one identified UDP-glucuronosyltransferase gene and the one glutathione S-transferase gene found in the midgut transcriptome of *A*. *compressa* (Tables 5 and 7), it can be assumed that these enzymes play a major role in neutralizing and/or minimizing the effects of xenobiotic compounds.

This study focused on DEGs significantly up- or down-regulated in the midgut of *A*. *compressa*. It thus gives a comprehensive assessment of digestive, detoxification, and stress-response-related genes that are putatively linked to transcriptional differences between life stages and/or feeding habits in *A*. *compressa*. For a comprehensive genome-wide comparison across other hymenopteran species, transcriptomic sequence data from the whole body and over all life stages need to be collected and analyzed. Our data might underrepresent the number of genes related to digestion, detoxification, and stress response identified in *A*. *compressa* when compared with other Hymenoptera genomes. However, the number of selected candidate genes coding for alpha-glucosidases, glycosyl hydrolase family 31, and cytochrome P450 was similar across all reference species and thus, might give a good indication on the number of genes identified in *A*. *compressa* (Table 9).

### Candidate gene expression between life stages of *A*. *compressa*

In this study, we found several DEGs in both life stages but with either differences in the expression levels (*i*.*e*. a higher or lower mean of normalized read counts) or regulation (S5 and S6 Tables and Fig 4). We found two genes coding for lipases and carbohydrases respectively, and eleven genes coding for diverse proteinases significantly up-regulated in both life stages (S5 and S6 Tables and Fig 4). The differentially expressed gene 15485 coding for a GNS1/SUR4 family, that is involved in long chain fatty acid elongation systems, was found up-regulated in the adult wasps with high expression levels but down-regulated in the larvae (Fig 4). Further, it should be noted that the gene g4146 coding for an aminotransferase class I and II was equally found down-regulated in both life stages (Fig 4). We also identified higher expression levels for a gene coding for the proteinase carboxypeptidase B-like in the adult wasps (Fig 4).

The most significantly up-regulated genes with higher expression levels in the adult wasps encode for the carbohydrase alpha-glucosidase (g16665) and glycosyl hydrolases family 31 (g13972) as shown in Fig 4. The latter shows a noticeably higher expression levels in the adult wasps than larvae (Fig 4). The high expression levels found among these carbohydrases might be related to an exclusively sugar-rich diet which we provided on a daily basis to the adult wasps. Due to this unlimited and essential food source, female wasps show intense reproduction behavior, with approximately two paralyzed cockroaches with oviposited eggs per day. It remains to be further investigated how sensitive the pattern of DEGs coding for carbohydrases and proteinases is going to change in natural conditions, *e*.*g*. if pollen and nectar are provided to the adult wasps in limited or unlimited quantities. Such changes might strongly affect the reproductive behavior and life span of both adult male and female wasps.

We found a higher amount of significantly up-regulated genes putatively related to detoxification, e.g. three cytochrome P450 and glutathion S-transferases (GSTs) in the midgut transcriptome of the adult wasps (Table 5 and S5 Table). While P450s modify residues of xenobiotic compounds to make them more hydrophilic, GSTs conjugate xenobiotic compounds to hydrophilic molecules [51]. As shown in the bumble bees *Bombus huntii* and *Melipona quadrifasciata*, adult females express higher levels of putative detoxification genes than adult males, and this in turn might be associated with haploidy and their activities being reduced to feeding themselves and mating [2, 52]. How and to what extent these detoxification enzymes are related to the metabolism of adult *A*. *compressa* also needs to be further investigated.

Larvae of *A*. *compressa* exhibit a notably high expression of several genes coding for proteinases (Fig 4). Particularly striking are the genes g14306 and g14307, which are classified as either chymotrypsin or trypsin (S2 Table). The second instar larva of *A*. *compressa* lives as an ectoparasite before entering the cockroachs’ body, and switching to an endoparasitoid life stage. Here, the larvae primarily feed on the cockroach’s hemolymph, which mainly consists of free amino acids, non-amino carboxylic acids and various carbohydrates [53]. Higher expression levels of specific DEGs coding for proteinases (Fig 4) might reflect a transcriptional response to a higher concentration of free amino acids in the cockroach’s hemolymph. On the other hand, proteases like trypsin and chymotrypsin are reported as good candidate enzymes to digest host tissue as shown in the ectoparasitoid larvae of *Euplectrus separatae* [54]. High levels of serine proteases in the midgut, such as chymotrypsin and tryspin, are reported in some other holometabolous larvae *e*.*g*., in the ant *Solenopsis invicta* [55] or the fruit fly *Ceratitis capitata* [56]. Further investigations with a higher sampling number of both the ectoparasitic and the endoparasitoid stages are needed to shed light onto transcriptional differences between life stages of *A*. *compressa*.

## Conclusion

The increasing number of complete insect genomes provides a crucial basis to understand various genomic adaptations, *e*.*g*., as related to nutrition, stress and toxic components response and behavior, especially when combined with transcriptomics [57]. In this study, we explored the midgut transcriptome of *A*. *compressa* to identify gut-biased DEGs with a putative role in digestion, detoxification and stress response. We identified 60 significant DEGs related to digestion, of which the two carbohydrases alpha-glucosidase and glycosyl hydrolase family 31 and the two proteinases chymotrypsin and trypsin exhibit the highest gene diversity.

We also identified six significant DEGs related to detoxification, including cytochrome P450s, GST, and UGT. The number of genes related to alpha-glucosidases, glycosyl hydrolases family 31, and cytochrome P450s is similar or lower than found in the nine reference Hymenoptera species. Differences in gene number, however, might be underestimates due to the analyzed transcriptomic dataset. Interestingly, we found no glycosyl hydrolases family 31 gene copy in any of the three reference bee species. Several DEGs exhibit considerably higher expression levels in the adult wasps than in the larvae, and *vice versa*. How and to what extent these observations hint at a transcriptional response related to life stage or dietary habit needs to be further investigated. The here identified digestive enzyme and detoxification genes provide a basis for future comparative genomic and proteomic studies, as well as for studies of functional evolution caused by (ontogenetic) dietary transitions in Hymenoptera.

## Supporting information

S1 Table Taxon sampling and sample preparation. Detailed list of all sampled individuals that were used for transcriptomic sequencing. All samples were collected at the Aquazoo Löbbecke Museum Düsseldorf, Germany. Provided are collection date, sex, life stage, and RNA extraction method and total RNA concentration.
(XLSX)

S2 Table Gut-biased differentially expressed genes (DEGs) coding for trypsin and chymotrypsin identified in *A*. *compressa*. Provided are up- (green) and down-regulated (red) DEGs identified for the adult wasps (AD) and larvae (LA) that correspond to chymotrypsin and trypsin. Enzymes are proofed according to their conserved cleavage site (CS) and the characteristic catalytic triad H-D-S (bold letters). Genes with no identified signal peptide and activation peptide motif were not included in the gene tree inference.
(XLSX)

S3 Table Sequencing, mapping statistics. Transcriptome sequencing statistics including data processing results of adapter and quality trimming as well as the mapping statistic obtained by HiSat2.
(XLSX)

S4 Table Gut-biased differentially expressed genes identified in *Ampulex compressa*. Provided are the following values for each gene: gene identifiers, mean normalized counts over all samples, log2 fold change, standard error estimate for the log2 fold change estimate, Wald statistic, p-value for the Wald statistic, *p*-value adjusted for multiple testing with the Benjamini-Hochberg procedure for the Wald statistic, Chromosome start and end, strand, and NCBI description. Genes labeled in green are counted as up- and genes labeled in red as down-regulated. The top 100 up- and down-regulated genes were searched against the NCBI database.
(XLS)

S5 Table Gut-biased differentially expressed genes (DEGs) with a putative role in digestion and detoxification identified in *A*. *compressa* adult wasps (DeSeq2 analysis A). Provided are up- (green) and down-regulated (red) DEGs. Functional annotation results identified by (1) NCBI Blast nr database search using blastp and (2) EggNog annotation. Furthermore, gene ontology (GO) classification on biological process, molecular function, and cellular component, and KEGG information are included.
(XLSX)

S6 Table Gut-biased differentially expressed genes (DEGs) with a putative role in digestion and detoxification identified in *A*. *compressa* larvae (DeSeq2 analysis A). Provided are up- (green) and down-regulated (red) DEGs. Functional annotation results identified by (1) NCBI Blast nr database search using blastp and (2) EggNog annotation. Furthermore, gene ontology (GO) classification on biological process, molecular function, and cellular component, and KEGG information are included.
(XLSX)

S7 Table Significant matches of alpha-glucosidase and glycosyl hydrolase family 31 genes identified in *A*. *compressa* and homologous genes searched across nine reference Hymenoptera species. Candidate genes identified in *A*. *compressa* were searched against the NCBI database and the top three NCBI Blast hits were further searched back against the genome of *A*. *compressa*. Only the top two hits were retained and counted as reliable match if they match the same scaffold and the same or similar position in the genome of *A*. *compressa* as the respective candidate genes. Provided are the enzyme name according to the pfam description and the number of identified gene copies.
(XLSX)

S8 Table Significant matches of cytochrome P450 genes identified in *A*. *compressa* and homologous genes searched across nine reference Hymenoptera species. Candidate genes identified in *A*. *compressa* were searched against the NCBI database and the top three NCBI Blast hits were further searched back against the genome of *A*. *compressa*. Only the top two hits were retained and counted as reliable match if they match the same scaffold and the same or similar position in the genome of *A*. *compressa* as the respective candidate genes. Provided are the enzyme name according to the pfam description and. the number of identified gene copies.
(XLSX)
